# An extensive re-evaluation of evidence and analyses of the Randomised Badger Culling Trial II: In neighbouring areas

**DOI:** 10.1098/rsos.240386

**Published:** 2024-08-21

**Authors:** Cathal L. Mills, Rosie Woodroffe, Christl A. Donnelly

**Affiliations:** ^1^ Department of Statistics, University of Oxford, Oxford, UK; ^2^ Pandemic Sciences Institute, University of Oxford, Oxford, UK; ^3^ Institute of Zoology, Zoological Society of London, London, UK

**Keywords:** epidemiology, badgers, tuberculosis, culling, cattle

## Abstract

In the second investigation in a pair of analyses which re-evaluates the Randomised Badger Culling Trial (RBCT), we estimate the effects of proactive badger culling on the incidence of tuberculosis (TB) in cattle populations in unculled neighbouring areas. Throughout peer-reviewed analyses of the RBCT, proactive culling was estimated to have detrimental effects on the incidence of herd breakdowns (i.e. TB incidents) in neighbouring areas. Using previously published, publicly available data, we appraise a variety of frequentist and Bayesian models as we estimate the effects of proactive culling on confirmed herd breakdowns in unculled neighbouring areas. For the during trial period from the initial culls until 4 September 2005, we estimate consistently high probabilities that proactive culling had adverse effects on confirmed herd breakdowns in unculled neighbouring areas, thus supporting the theory of heightened risk of TB for the neighbouring cattle populations. Negligible culling effects are estimated in the post-trial period across the statistical approaches and imply unsustained long-term effects for unculled neighbouring areas. Therefore, when considered alongside estimated beneficial effects within proactive culling areas, these conflicting adverse side effects render proactive culling complex, and thus, decision making regarding potential culling strategies should include (i) ecological, geographical and scientific considerations and (ii) cost–benefit analyses.

## 1. Introduction

### 1.1. Bovine tuberculosis in Great Britain

Bovine tuberculosis (TB) is an infectious zoonotic disease caused by *Mycobacterium bovis*. European badgers (*Meles meles*) have been identified as wildlife hosts for *M. bovis*. Due to the ecological, public health and economical consequences of bovine TB, badger culling strategies were implemented in attempts to curb transmission of *M. bovis* and thus reduce the incidence of TB in cattle across Great Britain.

In England, the Randomised Badger Culling Trial (RBCT) was a large-scale randomised field trial conducted in 30 trial areas (of similar size; approximately 100 km^2^) with high incidence of bovine TB. The RBCT was undertaken to examine the effectiveness of badger culling as a strategy for controlling incidence of TB in cattle in Great Britain. The trial was designed and overseen by a group of independent scientists, collectively known as the independent science group (ISG) on cattle TB [1]. The study design (including the ethical, ecological and statistical considerations) and associated culling activities of the RBCT were previously described in our first investigation [2].

The importance and policy relevance of an extensive assessment (of the effects of culling) within culled and unculled neighbouring areas are exemplified by the ongoing debates surrounding recent trends in bovine TB in Great Britain and the effectiveness of badger culling, as discussed in our first investigation [2].

### 1.2. Analyses of neighbouring areas of the RBCT

The statistical analyses of the RBCT were pre-defined (before the incidence data were collected) and also independently audited by a statistical auditor [1,3,4]. The response variables for the analyses were the observed (i) total number of herd breakdowns (inclusive of confirmed and unconfirmed) and (ii) number of confirmed herd breakdowns in a trial area. Confirmed herd breakdowns were herd incidents which involved evidence of TB exposure in at least one cattle herd member, and either lesion characteristic of TB was subsequently identified at post-mortem or the *M. bovis* organism was cultured. Otherwise, breakdowns were classified as unconfirmed.

An interim analysis of the RBCT was presented in a peer-reviewed analysis in 2003 [5]. The finding of increased TB incidence in cattle in areas subject to reactive culling led to the suspension of reactive culling. Subsequent ecological studies investigated the estimated increased TB risk induced by reactive culling and observed differences to badger ecology and behaviour as well as *M. bovis* epidemiology in badgers. In particular, increased ranges and increased mixing, and hence greater *M. bovis* transmission and prevalence, within badger populations, were estimated as well as increases to the number of cattle herds potentially in contact with each infected badger [6–9].

The peer-reviewed analyses of the RBCT consistently considered the effects of proactive culling on the incidence of herd breakdowns in the areas neighbouring proactive culling areas (alongside the within-culling areas). Consistent with the inferred effects of reactive culling, a 29% increased risk of TB, relative to herds in lands neighbouring unculled survey-only areas, was observed in cattle herds residing in neighbouring areas of up to 2 km outside the proactive culling areas [10]. Note that we analyse the effects of culling within proactive culling areas separately [2].

The ISG final report in 2007, which appraised 55.8 triplet years of the RBCT data, estimated that the beneficial effects of reduced TB incidence within proactively culled areas were offset by increased incidence in cattle in the unculled neighbouring areas [11]. Thus, due to the potential for contrasting effects of badger culling in the distinct areas, associated analyses explored the net effects of successive annual culls [12]. The estimated adverse effects in neighbouring areas tended to decline upon successive culls of the RBCT, and the estimated net effect was beneficial after the fourth and later annual culls. The reduced adverse effects over time in neighbouring areas were hypothesized to be a consequence of the changes to the badger population induced by repeated systematic culling. In particular, the culling was inferred to likely expand badgers’ daily ranges and increase their dispersal (the permanent movement from one location to another). Nevertheless, the short-distance nature of dispersal meant that the repeated culling was thought to be likely responsible for a depleted population of dispersers and achievement of a quasi-stable spatial organization [12].

Following the completion of the RBCT, post-trial analyses (from 1 year after the last cull) assessed whether the latter-stage results of reductions in elevated risk in neighbouring areas were sustained. The detrimental during-trial effects of greater TB incidence in neighbouring areas were estimated to be no longer present in an analysis of the post-trial period [13], and any beneficial post-trial effects disappeared after 18 months of post-culling [14]. A complementary analysis estimated that there were no sustained post-trial effects of proactive culling on incidence in the neighbouring areas up to March 2013 [15].

Finally, the findings and concerns from a recent preprint analysis by Torgerson *et al*. [16] were primarily related to the effects of badger culling within proactive culling areas and were individually addressed by Mills *et al*. [2]. With respect to the effects of culling in unculled neighbouring areas, the authors of [16] reported that, within a restricted period until 4 September 2005, an analysis displayed a lack of sufficient evidence for side effects of culling in the selected studied period and that the Poisson generalized linear model (GLM) approach taken by the ISG was inferior to alternative model formulations.

### 1.3. Objectives of the current analysis

The primary objective of the current analysis is to comprehensively re-assess the available evidence from the RBCT regarding the effects of proactive culling on herd breakdowns in unculled neighbouring areas. Our in-depth re-evaluation directly addresses the cited lack of evidence (proposed in the separate preprint manuscript by Torgerson *et al*. [16]) for the side effects in cattle populations in neighbouring areas.

We analyse the herd breakdowns data from three study periods (which were discussed in previous analyses of RBCT data): from the initial cull until 4 September 2005, from the first follow-up cull until 4 September 2005 and the post-RBCT trial period (from 1 year after the last cull until March 2013). Separately, our complementary analysis examines the effects of proactive culling within proactive culling areas across the same three study periods [2].

Our extension to a wide array of statistical techniques and study periods means that we extend beyond previous approaches taken by the ISG, by other subsequent analyses [17,18] and by the recent, separate preprint manuscript [16], and we subject each approach to the same rigorous model checking and model comparison. In doing so, we can make robust conclusions regarding the overall effects of proactive badger culling for neighbouring areas which are strongly informed by consistent scientific evidence, irrespective of which of the appropriate statistical approaches are taken.

## 2. Material and methods

### 2.1. Datasets and statistical considerations

The source of data for the current analysis is the RBCT, and our studied during-trial periods cover (i) from the *initial* proactive cull until 4 September 2005 and (ii) from the *follow-up* proactive cull until 4 September 2005; 4 September 2005 represents the last during-trial date for which trial data were available for the analysis by Donnelly *et al*. [10], while the recent preprint manuscript also used data from the initial cull until 4 September 2005 [16]. The during-trial data amount to totals of (i) 46.6 triplet years since the initial cull and (ii) 34.1 triplet years since the first follow-up cull. The post-trial period consists of 66.6 triplet years, from 1 year after the last cull until 28 March 2013. Our primary variable of interest is the number of *confirmed* herd breakdowns (or equivalently, the incidence) during the respective studied period in areas neighbouring proactive culling areas and in areas neighbouring survey-only areas (up to 2 km outside the culling areas).

For comprehensiveness, we also analyse incidence data from the *post-trial* period from 1 year after the last proactive cull until 28 March 2013, using data downloaded in July 2013. Confirmed herd breakdowns are recorded in 6-month time intervals.

In general, due to the statistical properties of our response (herd breakdowns represent count data), we note the potential for the phenomenon of overdispersion. The principles behind the manifestation of overdispersion, alongside various possible statistical approaches used to address the issue, are described at length in the accompanying analysis for within-culling areas [2].

### 2.2. Statistical methodology

#### 2.2.1. Statistical approach of the independent science group

The trial design of the RBCT, the statistical analyses of the independent science group (ISG) and the policies regarding the release of data were pre-defined and independently reviewed by a statistical auditor [1,3]. Regular, independent reviews by the statistical auditor continued from 2000 [4] until the ISG final report in 2007 [11,19].

The ISG-led, independently audited statistical analyses for the RBCT data involved the usage of a log-linear Poisson regression which modelled the observed herd breakdowns [1,3,5,10,12,20]. The Poisson regression model sought to quantify treatment/culling effects and made adjustments for factors such as individual triplet effects, the log of the number of baseline herds at risk and the log of the historic 3-year incidence of herd breakdowns. Alternative model formulations and sensitivity of inferences were appraised. Other formulations included covariate interactions with treatment effects, different time periods and adjustments for log of the baseline total cattle numbers and log of the total number of tests conducted. Results obtained were similar across each of the different modelling environments.

Our models estimate the effects of proactive culling by drawing comparison between the confirmed herd breakdowns in areas neighbouring proactive culling areas and in areas neighbouring survey-only areas. As previously described in [2], the models considered here enable a comprehensive assessment of the effects of proactive culling on confirmed herd breakdowns in neighbouring areas as the models again adopt differing parametric families (e.g. Poisson versus quasi-Poisson), specifications (e.g. covariate versus offset forms) and approaches to statistical inference (both frequentism and Bayesianism) across three studied periods (two during-trial periods and one post-trial period). The extension to a Bayesian perspective to modelling in each studied period (to make probabilistic statements about the effects of proactive culling) differs to the solely frequentist approaches taken by the ISG. Our separate, complementary analysis explores the number of confirmed herd breakdowns within proactive culling areas [2].

#### 2.2.2. Model appraisal and diagnostics

Across the frequentist and Bayesian settings, our model appraisal generally involves an in-depth assessment of the validity of underlying assumptions used to enable statistical inference. The model diagnostics are described elsewhere in [2], and we include a summary of key diagnostics employed for frequentist and Bayesian models in electronic supplementary material, tables S3 and S4, respectively.

## 3. Results

We present results here across frequentist and Bayesian statistical approaches, for data from the initial cull to 4 September 2005 and from the post-trial period (1 year after the last cull until March 2013). Results for incidence data from the first follow-up cull until 4 September 2005 are included in electronic supplementary materials, 4.2 and 5.2.

### 3.1. Frequentist approaches to modelling

#### 3.1.1. From the initial cull until 4 September 2005

The following analysis contains results from frequentist models fitted to confirmed herd breakdowns in areas neighbouring proactive culling areas and in areas neighbouring survey-only areas in the period from the initial culls of the RBCT until 4 September 2005. The incidence data analysed here are the same previously published, publicly available data from [10] and were also used in the recent, separate preprint manuscript by Torgerson *et al*. [16]. Table 1a,b contains a summary of our key results, and we subsequently provide an overview of key observations. Sample model diagnostics and checks are displayed in electronic supplementary material, 4.1.

**Table 1a.** For confirmed herd breakdowns from the initial cull until 4 September 2005 in areas neighbouring proactive culling areas and in areas neighbouring survey-only areas of the RBCT, we present a range of *frequentist* models. Various Poisson GLMs were examined across analyses of the RBCT led by the ISG, and a final Poisson GLM was fitted to the incidence data for the period from the initial cull until 4 September 2005 in [10]. The model was independently audited by a statistical auditor who certified the accuracy of the findings and the associated interpretations [19,21]. In a separate preprint manuscript by Torgerson *et al*. [16], other variants of the model were proposed, each of which are denoted by an asterisk (*) below. BIC and AICc denote the Bayesian information criterion and small-sample corrected Akaike information criterion, respectively. Lower values of each information criterion are better but note that it is not appropriate to compare the information criteria of the normal linear model (which assumes a continuous response and assumes normality of errors in a different model fitting method) with the Poisson-based models.

model	structure	estimated effect of culling (95% CI)	BIC	AICc	comments
1	original Poisson GLM (from [10])	28.8% (5.0%, 57.9%)	148.3	196.0	quantile deviation is detected in the simulated residual versus predicted plot in the quantile–quantile (QQ) plot of simulated residuals, the Kolmogorov–Smirnov test for uniformity of the scaled residuals and outlier test produce non-significant results from the test of dispersion and overdispersion for residuals, the deviation is significant (*p*‐value = 0.016) the residual versus predicted plot yields a non-significant result in the combined adjusted quantile test a visual posterior predictive check indicates that the model’s posterior predictive distribution resembles the observed data no influential observations are detected via leverage plots or Cook’s distance
2	quasi-Poisson GLM *	9.8% (−18.9%, 48.7%)	NA	NA	the lack of a defined probability distribution for the model likelihood prevents extensive model validation and checking via simulated residuals, information criteria or posterior predictive checks the model fit yields the lowest leave-one-out cross-validation (LOOCV) RMSE (root mean square error), although the model is outperformed in terms of LOOCV mean absolute error (MAE)(7.72) by model 8 (6.86). Information criteria cannot be used for the model no influential observations are identified due to Cook’s distance or hat values the quasi-Poisson model structure enables substantially increased flexibility such that the 95% CI for the estimated culling effect is wider and, thus, more uncertain
3	original GLM (from [10]) in generalized Poisson form	28.5% (14.2%, 44.7%)	143.8	217.2	across the checks of simulated residuals, the model does not produce any significant results which would indicate overdispersion or underdispersion, deviation, lack of uniformity or potential model misfit the posterior predictive check captures strong correspondence with the confirmed incidence data the LOOCV MAE (8.05) is inferior to the more flexible, quasi-Poisson model (model 2) fit (7.72)
4	generalized Poisson (without any culling effect) *	NA	154.5	153.2	relative to the models which estimate culling effects, the LOOCV MAE and RMSE are both inferior across the checks of simulated residuals, we report non-significant results for tests of overdispersion and underdispersion, outliers and lack of uniformity the posterior predictive check hints at a degree of potential model misspecification
5	generalized Poisson with herd-years-at-risk offset *	10.0% (−9.1%, 33.0%)	163.0	217.4	the LOOCV predictive accuracy is inferior to a model without a culling effect (such as model 4) and the original GLM of [10], both in terms of RMSE (18.66) and MAE (12.08) the posterior predictive check indicates potential model misspecification due to dissimilarities between model-simulated data and post-trial incidence no significant diagnostic results are attained across any of the checks of simulated residuals
6	generalized Poisson with herd-years-at-risk offset and without a culling effect *	NA	151.9	150.4	the tests of simulated residuals in the QQ plot all produce non-significant results for overdispersion or underdispersion, uniformity and deviations however, the combined adjusted quantile test (of the residual versus predicted plot) indicates significant deviations and, hence, potential simulation outliers potential model misspecification is captured by the posterior predictive check

**Table 1b.** For confirmed herd breakdowns from the initial cull until 4 September 2005 in areas neighbouring proactive culling areas and in areas neighbouring survey-only areas of the RBCT, we present a range of *frequentist* models. Various Poisson GLMs were examined across analyses of the RBCT led by the ISG, and a final Poisson GLM was fitted to the incidence data for the period from the initial cull until 4 September 2005 in [10]. The model was independently audited by a statistical auditor who certified the accuracy of the findings and the associated interpretations [19,21]. In a separate preprint manuscript by Torgerson *et al*. [16], other variants of the model were proposed, each of which are denoted by an asterisk (*) below. BIC and AICc denote the Bayesian information criterion and small-sample corrected Akaike information criterion, respectively. Lower values of each information criterion are better but note that it is not appropriate to compare the information criteria of the normal linear model (which assumes a continuous response and assumes normality of errors in a different model fitting method) with the Poisson-based models.

model	structure	estimated effect of culling (95% CI)	BIC	AICc	comments
7	generalized Poisson with herd-years-at-risk covariate *	28.5% (14.2%, 44.7%)	143.8	213.8	the posterior predictive check illustrates close correspondence between model-simulated data and the confirmed incidence data across the checks of simulated residuals, no significant overdispersion or underdispersion, outliers or departures from uniformity are detected the LOOCV analysis expectedly produces identical results to model 3 (when baseline herds at risk was used as a covariate instead of herd-years-at-risk)
8	generalized Poisson with herd-years-at-risk covariate without a culling effect *	NA	145.8	144.5	no significant dispersion, outliers or departures from uniformity are detected in the plots and tests of simulated residuals the lowest LOOCV MAE (6.86) among the frequentist model fits for the period from the initial cull until 4 September 2005 is attained for model 8, a model which does not account for any effect of culling in neighbouring areas and does not make assumptions about offset specification the model also yields the lowest AICc value (144.5) across any of the GLMs which share the common generalized Poisson likelihood however, the posterior predictive check indicates potential misspecification due to systematic discrepancies between the synthetic model-based predictions and the confirmed incidence data
9	normal linear model (incidence per herd year as response) *	1.5% (−1.2%, 4.2%)	−71.4	−31.2	the basic linear model quantifies a different response of incidence divided by herd-years-at-risk. Thus, CIs have a changed interpretation the key underlying assumption is a normal linear relationship between incidence per herd-years-at-risk and proactive culling. The modelling assumption is potentially restrictive and avoidable (in each study period), due to the conventional appropriateness of negative binomial and other GLM forms for potentially overdispersed count data the poor LOOCV predictive accuracy is characterized by the highest LOOCV MAE (12.29) for the period from the initial cull until 4 September 2005
10	normal linear model (incidence per herd year as response) without a culling effect *	NA	−79.0	−80.5	for the simple linear model without a culling effect, the assumption for normality of errors is potentially violated due to rejection of the Shapiro–Wilk test (*p*‐value < 0.01) for normality of residuals furthermore, the third highest LOOCV MAE (9.92) is attained for the linear model which does not account for any effect of proactive culling
11	generalized Poisson with only herd-years-at-risk as offset and no culling or triplet effects *	NA	151.2	149.9	based on the posterior predictive check ( electronic supplementary material, figure S4), the systematic discrepancies between the model-based predictions and the confirmed incidence hint at model misspecification for a model which assumes identical triplets the combined adjusted quantile test of the residual versus predicted plot indicates the potential presence of simulation outliers

From table 1a,b, we deduce that across a range of model fits, there is consistently an estimated adverse effect of proactive culling on the incidence of confirmed herd breakdowns in neighbouring areas. In three of the five GLMs which consider the side effects of proactive culling, the estimated effect is statistically significant. The two model exceptions are difficult to validate as being appropriate due to assumptions such as a highly flexible quasi-Poisson model structure or a possibly constraining assumption of an offset specification. Indeed, such model forms may induce potentially inappropriate model-based uncertainty or problematic diagnostic flaws.

No single model fit outperforms across all information criteria and predictive accuracy metrics. Nevertheless, the best-fitting GLM (model 8) in terms of several information criteria (such as corrected Akaike information criterion (AICc) which measures predictive capabilities) and several leave-one-out predictive accuracy metrics (which approximate model generalizability) is a model which does not account for any effect of culling on confirmed herd breakdowns in neighbouring areas. However, despite the representative fit of the model without any modelled culling effect, other model fits (such as the original Poisson GLM of [10] in generalized Poisson form) attain superior values for other information criteria such as Bayesian information criterion (BIC) (which is a measure of goodness of fit unlike Akaike information criterion and AICc which measures predictive accuracy).

#### 3.1.2. Post-trial period

We fitted the frequentist models to the confirmed herd breakdowns from the post-trial period, from 1 year after the last cull until 28 March 2013. Individual model appraisals are presented in electronic supplementary material, table S6a,b.

The results of the frequentist analysis for the post-trial period differ from the during-trial results as consistently, the estimated effects of culling in unculled neighbouring areas are negligible, with 95% CIs for the estimated effects concentrated symmetrically around small values near zero.

For instance, among the models which estimate the effect of proactive culling, under the best-fitting model (in terms of leave-one-out predictive accuracy and information criteria such as AICc and BIC), the estimated culling effect is marginally beneficial and not statistically significant (estimate: −3.0%, 95% CI: −16.7%, 12.9%).

Indeed, no model fit for the post-trial period yields a significant (either beneficial or detrimental) estimated effect of proactive culling. Hence, we observe superior model fits for model structures which do not account for any post-trial effects of culling on the incidence of confirmed herd breakdowns in neighbouring areas.

### 3.2. Bayesian approaches to modelling

To enhance the robustness of model-based inferences and reduce sensitivity to the modelling approach used, we now present results from Bayesian models fitted to confirmed herd breakdowns in areas neighbouring proactive culling areas and in areas neighbouring survey-only areas.

#### 3.2.1. From the initial cull until 4 September 2005

**Figure 1.**
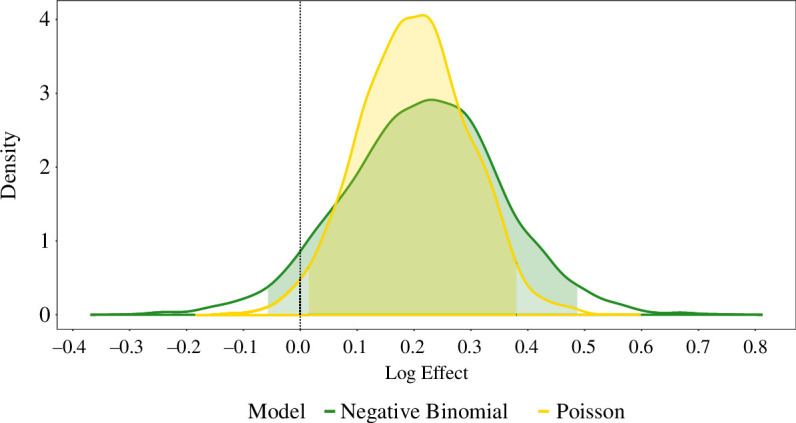
The *posterior distribution*, alongside 95% CrI, of the treatment effect from the *best-fitting Bayesian negative binomial* (Model c.2; green) *and Poisson* (Model e; yellow) GLMs fitted to confirmed herd breakdowns for the *period from the initial cull until 4 September 2005*. The vertical dashed line located at zero is reflective of hypothetical absence of any culling effect. From the posterior distributions, we deduce that under the models’ conditions, there are probabilities of 93.2% and 98.3%, respectively, that the proactive culling effect on confirmed incidence was detrimental to unculled neighbouring areas in the period from the initial cull until 4 September 2005.

Table 2a,b captures the key results from Bayesian models for the period from the initial cull until 4 September 2005, and we subsequently summarize observations from across the models. In figure 1, we visualize the implied effect of culling for neighbouring areas under the best-fitting Bayesian negative binomial and Poisson GLMs. Noteworthy sample model diagnostics and checks are displayed in electronic supplementary material, 5.1. Posterior predictive-based diagnostics enable identification of systematic model misspecification, comparison of model fits and, hence, conclusions regarding model appropriateness using varying forms of likelihood and varying prior distributions.

**Table 2a.** For confirmed herd breakdowns from the initial cull until 4 September 2005 in areas neighbouring proactive culling areas and in areas neighbouring survey-only areas of the RBCT, we present a range of Bayesian models. The original, frequentist Poisson GLM used in [10] was re-specified in the Bayesian paradigm in the separate preprint manuscript by Torgerson *et al*. [16], with an alternative negative binomial likelihood (as opposed to Poisson likelihood), and is labelled as Models a.1 and a.2 below. Models discussed in the separate preprint manuscript by Torgerson *et al*. [16] are denoted by an asterisk (*), and our improved/alternative model versions are denoted by two asterisks (**). With respect to the preprint manuscript by Torgerson *et al*. [16], we employ a different labelling system for models, and the corresponding label from the preprint manuscript can be found in electronic supplementary material, table S2. Estimated culling effects and associated uncertainty are reported using the posterior median and 95% credible intervals (CrIs) of exponentiated posterior samples of the models’ culling effect parameter.

model	estimated effect of culling (95% CrI)	LOO ELPD	diagnostics	conclusions
a.1 (varying intercepts for triplets and covariates of culling effect, historical 3-year incidence and baseline herds at risk) *	31.3% (−29.4%, 160.9%)	−85.0	the posterior predictive checks, across density-based visualizations and distribution of maxima, indicate severe model misfit, and the model’s extreme posterior predictions are too implausibly large for the confirmed incidence data the implausibility of model-based predictions is an apparent consequence of the strongly informative prior distribution assumption for dispersion which yields extreme overdispersion (electronic supplementary material, figures S11 and S12) consequently, the sample average of the posterior predictive distribution (36.4) is considerably greater than the mean number of confirmed herd breakdowns (28.5)	under the conditions of the model, there is an 80.7% probability that proactive culling had an adverse effect on confirmed incidence in neighbouring areas, although the extreme bounds of the 95% CrI are perhaps inappropriate due to the uncovered diagnostic flaws of the model
a.2 (a.1 improved)**	24.5% (−3.6%, 69.9%)	−74.0	model fit, in terms of estimated out-of-sample predictive accuracy (measured by LOO ELPD) and, thus, generalizability of the model, is improved by specifying only weakly informative prior distributions the prior distributions specified include a heavier-tailed, less informative, half-Cauchy prior distribution for the auxiliary parameter (reciprocal dispersion) and a scientifically plausible normal (0,1) prior distribution for the treatment effect furthermore, the sample average posterior predictive distribution (29.2) is far closer to the mean confirmed incidence (28.5) however, while the density-based posterior predictive check improves upon the previous model’s fit, potential model misspecification remains due to the presence of occasional, extreme incidence predictions	under the conditions of the model, there is a 95.9% probability that proactive culling had an adverse effect on confirmed incidence in neighbouring areas, although the implied extreme adverse effects (captured by large positive values in the 95% CrI) likely remain implausible due to the model’s misspecification issues
b.1 (no varying intercepts for triplets) *	25.6% (−19.4%, 97.8%)	−74.9	the attained LOO ELPD value and the posterior predictive check indicate superior model fit (to the confirmed incidence data) than previous (unimproved) models for the period from the initial cull until 4 September 2005 however, the graphical posterior predictive check hints at potential model misspecification due to the presence of a small number of large, implausible posterior predictions	under the conditions of the model, there is an 84.5% probability that the proactive culling effect on confirmed incidence is detrimental for areas neighbouring proactive culling areas
b.2 (b.1 improved)**	24.3% (−5.5%, 64.1%)	−70.8	the specification of weakly informative prior distributions once again produces superior estimated out-of-sample predictive accuracy (measured by LOO ELPD) and, hence, greater generalizability of model-based inferences similarly, the better in-sample model fit is captured by the posterior predictive check which removes the presence of extreme, implausibly large posterior predictions	under the conditions of the model, there is a 94.0% probability that the proactive culling effect was adverse for incidence in neighbouring areas

**Table 2b.** For confirmed herd breakdowns from the initial cull until 4 September 2005 in areas neighbouring proactive culling areas and in areas neighbouring survey-only areas of the RBCT, we present a range of Bayesian models. The original, frequentist Poisson GLM used in [10] was re-specified in the Bayesian paradigm in the separate preprint manuscript by [16], with an alternative negative binomial likelihood (as opposed to Poisson likelihood), and is labelled as Models a.1 and a.2. Models discussed in the separate preprint manuscript by Torgerson *et al*. [16] are denoted by an asterisk (*), and our improved/alternative model versions are denoted by two asterisks (**). With respect to the preprint manuscript by Torgerson *et al*. [16], we employ a different labelling system for models, and the corresponding label from the preprint manuscript can be found in electronic supplementary material, table S2. Estimated culling effects and associated uncertainty are reported using the posterior median and 95% credible intervals (CrIs) of exponentiated posterior samples of the models’ culling effect parameter.

model	estimated effect of culling (95% CrI)	LOO ELPD	diagnostics	conclusions
c.1 (using herd-years-at-risk as an offset and no varying intercepts for triplets) *	25.8% (−19.5%, 97.2%)	−74.8	the graphical posterior predictive check hints at potential model misspecification	under the conditions of the model, there is an 85.0% probability that the proactive culling effect is detrimental to confirmed herd breakdowns in neighbouring areas
c.2 (c.1 improved)**	23.6% (−6.0%, 67.3%)	−70.5	the specification of weakly informative prior distributions enables a slightly superior model fit, as captured by the closer correspondence between posterior predictions and the confirmed incidence data and the removal of extreme, implausible posterior predictions superior model fit is also displayed by the estimated out-of-sample predictive accuracy (measured by the LOO ELPD)	the model fit indicates that the best-fitting negative binomial model (according to leave-one-out predictive accuracy and posterior predictive checking) yields a 93.2% probability of an adverse culling effect on confirmed incidence in neighbouring areas
d.1 (no culling effect) *	NA	−74.8	the posterior predictive check implies model misspecification due to discrepancies between model-based posterior predictions and the confirmed incidence data, as well as the presence of a number of implausibly large predictions	the model does not facilitate any direct probability statements about the effect of proactive culling on confirmed incidence in neighbouring areas
d.2—no culling effect (d.1 improved) **	NA	−71.2	the improved model fit is captured by the posterior predictive distribution, which closely resembles the observed data and does not contain the implausibly large synthetic model-based predictions furthermore, the estimated out-of-sample predictive accuracy (measured by LOO ELPD) and, hence, the generalizability of the model are improved	while the improved model structure does not enable direct, probabilistic inferences about the size of the effect of proactive culling, the lack of any discernible model diagnostic flaws is indicative of the appropriateness of the model (which does not account for any effect of culling)
e (Poisson with baseline herds at risk as an offset) **	26.6% (4.3%, 52.8%)	−70.0	the visual posterior predictive check indicates potential misspecification due to the systematic discrepancies between the posterior predictions and the confirmed incidence despite the varying likelihood form, the attained LOO ELPD value can be directly compared to the other Bayesian negative binomial GLMs, and thus, the Poisson GLM indicates superior implied out-of-sample predictive accuracy similarly, the posterior predictive checks, both in terms of density-based visualizations and distribution of the maximum incidence, indicate that our model recovers more plausible posterior predictions of incidence	under the Bayesian Poisson GLM, there is a 98.3% probability that the proactive culling effect on confirmed incidence in neighbouring areas was adverse (figure 1)

Under the conditions of various Bayesian GLMs with weakly informative, regularizing prior distributions, there are high probabilities of between 93.2% and 98.3% that proactive culling has an adverse effect on the incidence of confirmed herd breakdowns in areas neighbouring proactive culling areas and in areas neighbouring survey-only areas. Potential model misspecification is implied by the fitted negative binomial models from the separate preprint manuscript by Torgerson *et al*. [16]. Hence, focusing on our proposed improved models which yield greater estimated generalizability, under the best-fitting negative binomial GLM (Model c.2), there is a 94.0% probability that proactive culling had an adverse effect on confirmed herd breakdowns in neighbouring areas.

Interestingly, a model (Model d.2) without any explicit consideration for an effect of culling does not yield any discernible model diagnostic flaws, which is perhaps reflective of uncertainty regarding the extent of the effect of proactive culling on herd breakdowns in neighbouring areas. Nevertheless, the findings are consistent with those of [10] as the range of best-fitting models implies that it is highly probable that proactive culling resulted in increased incidence in adjoining or neighbouring areas in the period from the initial proactive cull until 4 September 2005.

#### 3.2.2. Post-trial period

**Figure 2.**
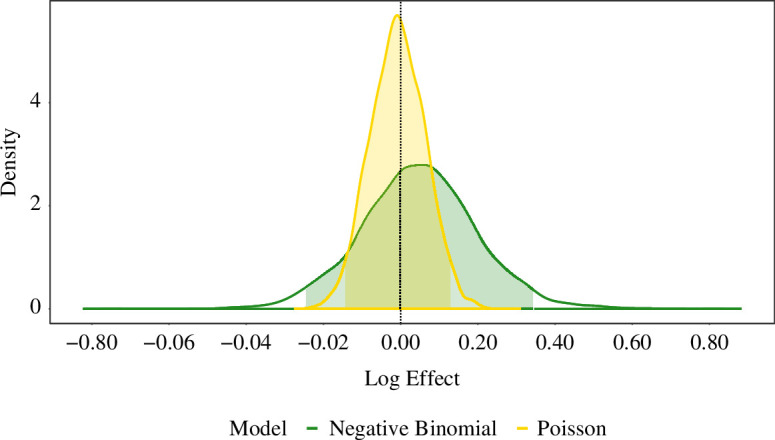
Among the models which explicitly consider culling effects, the *posterior distribution*, alongside 95% CrI, of the *treatment effect parameter* from the *best-fitting Bayesian negative binomial* (Model c.2; green) *and Poisson* (Model e; yellow) GLMs fitted to confirmed herd breakdowns for the *post-trial period* (from 1 year after the last proactive cull until 28 March 2013). The vertical dashed line located at zero is reflective of hypothetical absence of any culling effect. From the posterior distributions, we deduce that under the negative binomial and Poisson models’ conditions, there are 63.2% and 45.3% probabilities, respectively, that the proactive culling effect on confirmed incidence was adverse, yet there are high probabilities of 74.5% and 98.2%, respectively, that the effect was less than a 15% increase on confirmed incidence.

We fitted the Bayesian models to confirm herd breakdowns in the post-trial period, from 1 year after the last proactive cull until 28 March 2013. Individual model appraisals are presented in electronic supplementary material, table S8a–c, where the same suite of model diagnostics are employed, identical to those performed for the two during-trial study periods. Figure 2 illustrates the implied culling effects under the best-fitting Bayesian negative binomial and Poisson GLMs.

Similar to our frequentist-based analysis, across a range of Bayesian models for the post-trial period (electronic supplementary material, table S8a–c) [22–24], we estimate small and negligible post-trial effects of proactive culling on confirmed incidence in neighbouring areas. In particular, under the conditions of any of the models, the estimated 95% credible intervals (CrIs) for the effects of culling are uncertain and almost symmetric around zero. Among the subset of models which explicitly account for any effects of proactive culling, under the best-fitting model (figure 2), there is a 63.2% probability (Model c.2) that proactive culling had a detrimental effect on confirmed herd breakdowns in neighbouring areas, yet there is a 74.5% probability that the adverse effect was less than a 15% increase in confirmed herd breakdowns.

Indeed, as a consequence of the estimated negligible effects of proactive culling on confirmed herd breakdowns, we again find (consistent with the separate frequentist analysis for the post-trial period) that a model without any consideration for the effects of proactive culling (Model d.2) achieves the best (estimated) out-of-sample predictive accuracy. The superiority of a model structure without a model proactive culling effect is apparently due to the unsustained nature of any significant culling effects on herd breakdowns in neighbouring areas.

## 4. Discussion

We have comprehensively re-assessed the available evidence regarding the effects of proactive culling on the incidence of herd breakdowns for cattle residing in unculled neighbouring areas. Similar to our accompanying analysis [2], our modelling re-evaluation covers three study periods (two during-trial and one post-trial), and across two distinct approaches to statistical inference (frequentism and Bayesianism), a wide range of model frameworks (identical to [2] to ensure consistency) are analysed.

In the period from the initial cull until 4 September 2005 (the period previously analysed by [10] and discussed in the recent separate preprint manuscript by [16]), across various appropriate frequentist models, we estimated adverse effects of proactive culling on the incidence of confirmed herd breakdowns in areas neighbouring culled areas. The implied adverse effect was consistently significant across several well-fitting models, and the finding aligns with the conclusions of various peer-reviewed analyses. The estimated adverse effect was subject to a degree of modelling uncertainty as a model structure without explicit consideration of the effects of proactive culling achieved a representative fit (in terms of some out-of-sample predictive accuracy metrics and some information criteria). Therefore, our extension to a separate Bayesian analysis allowed us to directly address any uncertainty regarding the effects of proactive culling for unculled neighbouring areas. The Bayesian models consistently assigned high probabilities to adverse effects of culling on herd breakdowns in areas neighbouring proactively culled areas, relative to areas which neighbour survey-only areas. For instance, under the conditions of the best-fitting, most generalizable Bayesian model (Model c.2), there is a 93.2% probability that proactive culling had an adverse effect on confirmed herd breakdowns in neighbouring areas.

Expanding our focus to the period from the first follow-up proactive cull until 4 September 2005, irrespective of the distinct approaches to statistical inference (either frequentist or Bayesian models), we again estimated with high probabilities that there were detrimental side effects of proactive culling for cattle in neighbouring areas. Nevertheless, the best-fitting frequentist model fit (for the period from the first follow-up cull until 4 September 2005) was attained for a structure which did not account for the effect of proactive culling. The model fit was superior across a range of out-of-sample predictive metrics and information criteria, which is perhaps a consequence of decreasingly substantial side effects of proactive culling in a restricted study period that focuses more heavily on the latter stages of the during-trial period. Our Bayesian analysis still assigned high plausibility to the event that proactive culling had adverse effects for cattle populations in neighbouring areas, albeit estimating weaker overall effects. The weaker detrimental effects of proactive culling (for cattle in neighbouring areas) from the first follow-up cull until 4 September 2005 (relative to the period from the initial cull until 4 September 2005) had been observed by separate, peer-reviewed analyses of the RBCT which employed different statistical methods [10,12]. The phenomenon is apparently a consequence of the net beneficial effects of individual, latter-stage proactive culls, in contrast to the marked net detrimental effect between the initial and first follow-up cull [12].

In the post-trial period, our range of frequentist models consistently estimated negligible (non-significant) and, thus, non-existent effects of proactive culling on herd breakdowns for unculled neighbouring areas. Indeed, the best-fitting, most generalizable models were consistently those that did not explicitly consider any post-trial effect of proactive culling for the neighbouring areas. The findings of non-existent and, thus, unsustained effects of proactive culling in neighbouring areas were substantiated and quantified more precisely by our separate Bayesian model-based estimates of high probabilities for limited post-trial effects of culling.

Therefore, based on our comprehensive analyses, we deduce that during the RBCT, irrespective of statistical methodology, proactive culling was associated with a heightened risk (albeit declining with time) of herd breakdowns for cattle residing in areas neighbouring culled areas, compared to cattle populations in areas neighbouring unculled survey-only areas. The adverse side effects of proactive culling, particularly in the immediate aftermath of the first proactive cull, represent important considerations for scientists and policy-makers during the design and implementation of any potential badger culling strategy. Consequently, it may be required to perform comprehensive assessments of the geographies of any planned culling areas and their environs. Furthermore, our analysis reveals that the side effects of proactive culling for neighbouring areas declined over time and were unsustained following the completion of annual culls, thus apparently removing concerns regarding long-term effects in the absence of concurrent proactive culls. Indeed, the estimated during-trial adverse effects for neighbouring areas were substantiated by the absence of adverse effects in neighbouring areas when proactive culling was removed. Therefore, we deduce that any proactive culling strategies require careful consideration for neighbouring areas during culling due to adverse effects between annual culls, yet in contrast to the post-trial findings for within-culling areas, post-trial considerations are apparently not as important for neighbouring areas as there are no long-term effects beyond the final annual cull.

Combining the analysis with the beneficial during-trial and post-trial effects for cattle populations within proactive culling areas [2], we conclude that development of any proactive culling strategy is likely to remain controversial and require in-depth ecological and scientific considerations. It would be instructive to carefully consider the estimated benefits to cattle residing within proactive culling areas alongside the consideration of risk mitigation strategies for neighbouring cattle populations. A detailed cost–benefit analysis would be a suitable avenue to scrutinize such contrasting estimated benefits and risks to proactively culled and unculled neighbouring areas, respectively.

## Data Availability

All code and data used to produce our analysis are available at [22] and at Zenodo [23]. Supplementary material is available online [24].

## References

[1] Bourne J , Donnelly CA , Gettinby G , McInerney JP , Morrison WI , Woodroffe R , Cox DR . 1998 Towards a sustainable policy to control TB in cattle – a scientific initiative. First report of the independent scientific group on cattle TB. See https://webarchive.nationalarchives.gov.uk/ukgwa/20081023164230/http://www.defra.gov.uk/animalh/tb/isg/isgrep1.htm.

[2] Mills CL , Rosie W , Christl AD . 2024 An extensive re-evaluation of evidence and analyses of the Randomised Badger Culling Trial (RBCT) I: Within proactive culling areas. R. Soc. Open Sci. (10.1098/rsos.240385) PMC11335396 39169965

[3] Bourne J , Donnelly CA , Gettinby G , McInerney JP , Morrison WI , Woodroffe R , Cox DR . 2000 Notes on statistical aspects of the badger culling trial http://www.defra.gov.uk/animalh/tb/publications/progress/isganna.htm

[4] Mollison D . 2000 First report of the statistical auditor on the badger culling trial. See https://webarchive.nationalarchives.gov.uk/ukgwa/20081024050611/http://www.defra.gov.uk/animalh/tb/publications/auditor/stats1.htm.

[5] Donnelly CA , Woodroffe R , Cox DR , Bourne J , Gettinby G , Le Fevre AM , McInerney JP , Morrison WI . 2003 Impact of localized badger culling on tuberculosis incidence in British cattle. Nature **426** , 834–837. (10.1038/nature02192) 14634671

[6] Woodroffe R *et al* . 2005 Effects of culling on badger meles meles spatial organization: implications for the control of bovine tuberculosis: effects of culling on badger spatial organization. J. Appl. Ecol. **43** , 1–10. (10.1111/j.1365-2664.2005.01144.x)

[7] Woodroffe R *et al* . 2006 Culling and cattle controls influence tuberculosis risk for badgers. Proc. Natl Acad. Sci. USA **103** , 14713–14717. (10.1073/pnas.0606251103) 17015843 PMC1586183

[8] Woodroffe R *et al* . 2009 Bovine tuberculosis in cattle and badgers in localized culling areas. J. Wildl. Dis. **45** , 128–143. (10.7589/0090-3558-45.1.128) 19204342

[9] Ham C , Donnelly CA , Astley KL , Jackson SYB , Woodroffe R . 2019 Effect of culling on individual badger Meles meles behaviour: potential implications for bovine tuberculosis transmission. J. Appl. Ecol. **56** , 2390–2399. (10.1111/1365-2664.13512) 34565831 PMC8447922

[10] Donnelly CA *et al* . 2006 Positive and negative effects of widespread badger culling on tuberculosis in cattle. Nature **439** , 843–846. (10.1038/nature04454) 16357869

[11] Bourne J *et al* . 2007 Bovine TB: the scientific evidence. A science base for A sustainable policy to control TB in cattle. final report of the independent scientific group on cattle TB.

[12] Donnelly CA *et al* . 2007 Impacts of widespread badger culling on cattle tuberculosis: concluding analyses from a large-scale field trial. Int. J. Infect. Dis. **11** , 300–308. (10.1016/j.ijid.2007.04.001) 17566777

[13] Jenkins HE , Woodroffe R , Donnelly CA . 2008 The effects of annual widespread badger culls on cattle tuberculosis following the cessation of culling. Int. J. Infect. Dis. **12** , 457–465. (10.1016/j.ijid.2008.04.001) 18502675

[14] Jenkins HE , Woodroffe R , Donnelly CA . 2010 The duration of the effects of repeated widespread badger culling on cattle tuberculosis following the cessation of culling. PLoS One **5** , e9090. (10.1371/journal.pone.0009090) 20161769 PMC2818840

[15] Donnelly CA . 2013 Results from the Randomised Badger Culling Trial based on data downloaded in July 2013. See https://www.bovinetb.info/docs/results-from-the-randomised-badger-culling-trial-based-on-data-downloaded-in-july-2013.pdf.

[16] Torgerson P , Hartnack S , Rasmusen P , Lewis F , Langton T . 2022 Absence of effects of widespread badger culling on tuberculosis in cattle. In Review. (10.21203/rs.3.rs-2362912/v1) PMC11251155 39009688

[17] Mill AC , Rushton SP , Shirley MDF , Murray AWA , Smith GC , Delahay RJ , McDonald RA . 2012 Farm-scale risk factors for bovine tuberculosis incidence in cattle herds during the randomised badger culling trial. Epidemiol. Infect. **140** , 219–230. (10.1017/S0950268811000434) 21439101

[18] Karolemeas K , Donnelly CA , Conlan AJK , Mitchell AP , Clifton-Hadley RS , Upton P , Wood JLN , McKinley TJ . 2012 The effect of badger culling on breakdown prolongation and recurrence of bovine tuberculosis in cattle herds in Great Britain. PLoS One **7** , e51342. (10.1371/journal.pone.0051342) 23236478 PMC3517421

[19] Mollison D . 2007 Final report of the statistical auditor on the badger culling trial. See https://webarchive.nationalarchives.gov.uk/ukgwa/20081024050611/http://www.defra.gov.uk/animalh/tb/publications/auditor/stats1.htm.

[20] Mollison D . 2005 Statistical aspects of the Randomised Badger Culling Trials: Report for 2004/5. See https://webarchive.nationalarchives.gov.uk/ukgwa/20081024050733mp_/http://www.defra.gov.uk/animalh/tb/culling/rbct-report.pdf.

[21] Mollison D . Audit of Report by ISG to the Minister on Interim Analyses of RBCT Data. Technical report, September 2005. See https://webarchive.nationalarchives.gov.uk/ukgwa/20070814120000/http:/www.defra.gov.uk/animalh/tb/isg/news-archive.htm.

[22] Mills C . rcbt analyses. GitHub. See https://github.com/cathalmills/rbct_analyses/.

[23] Mills C . 2024 Cathalmills/rbct_analyses: RBCT analyses release pre-publication. Zenodo. See https://zenodo.org/doi/10.5281/zenodo.11527588.

[24] Mills C , Donnelly CA , Woodroffe R . 2024 Data from: An extensive re-evaluation of evidence and analyses of the Randomised Badger Culling Trial (RBCT) II: Neighbouring areas. Figshare. (10.6084/m9.figshare.c.7313751) PMC11335398 39169967

